# 凡德他尼治疗非小细胞肺癌的*meta*分析

**DOI:** 10.3779/j.issn.1009-3419.2012.03.07

**Published:** 2012-03-20

**Authors:** 玲 陶, 文磊 卓, 帆 杨, 波 朱

**Affiliations:** 400037 重庆，第三军医大学新桥医院全军肿瘤研究所 Institute of Cancer, Xinqiao Hospital, the Third Military Medical University, Chongqing 400037, China

**Keywords:** 凡德他尼, 肺肿瘤, *Meta*分析, Vandetanib, Lung neoplasms, *Meta*-analysis

## Abstract

**背景与目的:**

凡德他尼是抑制血管内皮生长因子受体（vascular endothelial growth factor receptor, VEGFR）和内皮生长因子受体（endothelial growth factor receptor, EGFR）的小分子药物，本研究旨在系统评价凡德他尼作为二线方案治疗非小细胞肺癌（non-small cell lung cancer, NSCLC）的有效性和安全性。

**方法:**

检索PubMed、Medline、Embase、维普、中国期刊全文数据库等数据库，收集凡德他尼治疗NSCLC的随机对照试验，用Revman 5.0软件对数据进行*meta*分析。

**结果:**

与对照组（单用一种其它靶向药物或化疗药物）相比，凡德他尼组在疾病无进展生存期（OR=1.23, 95%CI: 1.05-1.45）、部分缓解（OR=2.15, 95%CI: 1.59-2.93）、疾病控制（OR=1.22, 95%CI: 1.06-1.40）、腹泻（OR=1.59, 95%CI: 1.38-1.83）、恶心（OR=0.69, 95%CI: 0.57-0.83）、皮疹（OR=2.07, 95%CI: 1.71-2.49）、便秘（OR=0.81, 95%CI: 0.67-0.97）、呕吐（OR=0.72, 95%CI: 0.60-0.87）等方面有统计学差异，但总生存期、疾病稳定、疲乏、咳嗽、食欲减退、呼吸困难等方面无统计学差异。

**结论:**

凡德他尼作为二线方案治疗晚期NSCLC的疗效有一定的优势，但是其安全性无明显优势。

## Abstract (en)

**Background and objective:**

Vandetanib is a small molecule inhibitor against vascular endothelial growth factor receptor (VEGFR) and epidermal growth factor receptor (EGFR). The aim of this study is to evaluate the efficacy and safety of vandetanib as a second-line treatment for advanced non-small cell lung cancer (NSCLC).

**Methods:**

We selected randomized controlled trials (RCTs) on vandetanib for NSCLC from PubMed, Medline, Embase, VIP and CNKI. *Meta*-analysis was completed using software Review Manager 5.0.

**Results:**

Compared with the control group (single other targeted therapy or chemotherapy group), there were statistical differences in progression free survival (PFS) (OR=1.23, 95%CI: 1.05-1.45), partial response (PR) (OR=2.15, 95%CI: 1.59-2.93), disease control (DC) (OR=1.22, 95%CI: 1.06-1.40), diarrhea (OR=1.59, 95%CI: 1.38-1.83), nausea (OR=0.69, 95%CI: 0.57-0.83), rash (OR=2.07, 95%CI: 1.71- 2.49), constipation (OR=0.81, 95%CI: 0.67-0.97), and vomiting (OR=0.72, 95%CI: 0.60-0.87) in the vandetanib group, but there were no differences in overall survival (OS), stable disease (SD), fatigue, cough, anorexia and dyspnea.

**Conclusion:**

Vandetanib might have more superior efficacy as a second-line treatment for NSCLC, but its advantages in terms of safety were not demonstrated.

肺癌是当今威胁人类健康的死因之一，其中75%-80%是非小细胞肺癌（non-small cell lung cancer, NSCLC）^[1]^，包括腺癌、鳞状细胞癌、大细胞癌等。大部分患者确诊时已属晚期^[2]^，化疗仍是NSCLC治疗的重要手段。许多患者经一线方案治疗后能够保持完全或部分缓解，但仍有绝大部分患者表现为疾病进展甚至死亡。一些新的靶向药物作为二线方案治疗NSCLC已被逐渐接受^[3-8]^，包括多西他赛、培美曲塞、厄洛替尼、吉非替尼。但是这些药物尚未显示出明确的优势，需要发展新的治疗策略^[9]^。

凡德他尼是近年来研发的一种抑制血管内皮生长因子受体（vascular epidermal growth factor receptor, VEGFR）和表皮生长因子受体（epidermal growth factor receptor, EGFR）信号通路的分子靶向药物，也是RET（rearranged during transfection）受体酪氨酸蛋白激酶抑制剂^[10]^。2006年1月凡德他尼获欧洲罕见病药品委员会批准，推荐用于治疗髓样甲状腺癌^[11]^、乳腺癌、骨髓瘤等^[12]^。凡德他尼用于治疗西方^[13]^和日本^[14]^晚期肿瘤患者的Ⅰ期临床试验显示，凡德他尼的耐受剂量是300 mg/d。同时日本的研究^[14]^发现9例晚期NSCLC患者中有4例有抗肿瘤效果。这一结果促进了凡德他尼治疗NSCLC的临床研究，已有随机对照试验显示凡德他尼可作为一线^[15]^、二线^[16]^方案治疗晚期NSCLC。

凡德他尼作为二线方案治疗NSCLC的临床效应尚存在争议，凡德他尼加多西他赛与单用多西他赛的Ⅲ期临床对照研究^[3]^报道，凡德他尼可提高疾病无进展生存期。但是另一项Ⅲ期临床研究^[9]^显示，凡德他尼无相应的临床效应优势。为了进一步证实凡德他尼作为二线方案治疗NSCLC的疗效和安全性，本研究运用*meta*分析的方法对其临床随机对照试验进行综合分析，以期为晚期NSCLC治疗提供依据。

## 1 资料与方法

### 1.1 纳入与排除标准

纳入标准：①研究设计：随机对照研究；②研究对象：年龄≥18岁，经病理/细胞学检查证实的晚期NSCLC患者；③干预措施：凡德他尼组*vs*对照组（单用一种其它靶向药物或化疗药物），不限制凡德他尼和其它药物的剂量和疗程；④结局指标：总生存期、无进展生存期、副作用（腹泻、便秘、恶心等）。排除标准：①无法获得全文的会议摘要；②Jadad评分在3分以下的文章；③干预措施为非随机对照试验；④合并有小细胞肺癌或其它恶性肿瘤、肝肾功能损害者。

### 1.2 检索策略

计算机检索PubMed、Medline、Embase、Cochrane协作网、维普、中国期刊全文数据库等中外数据库。检索词包括非小细胞肺癌（non-small cell lung cancer, NSCLC）、凡德他尼（vandetanib）、随机对照试验（randomized controlled trials, RCTs）等。因凡德他尼治疗NSCLC的Ⅰ期临床研究开始于2002年^[17]^，故文献检索时间为2000年1月-2011年9月，同时手工检索相关期刊，必要时向有关专家和公司索要有关研究资料。

### 1.3 文献筛选和资料提取

两位研究者交叉核对纳入研究结果，对有分歧的意见与第3位研究者讨论后决定是否纳入，摘录内容包括：①一般资料：题目、作者姓名、发表日期、病例数；②研究特征：研究对象的一般情况、干预措施；③有效性结局指标：总生存期（overall survival, OS）、无进展生存期（progression free survival, PFS）、部分缓解（partial responses, PR）疾病控制（disease control, DC）、疾病稳定（stable disease, SD）；④安全性结局指标：腹泻、便秘、疲乏、皮疹、恶心、咳嗽、食欲减退、呼吸困难、呕吐。

### 1.4 质量评价

按照Jadad评分评价随机对照研究的质量，由两位评价员独立对纳入研究的随机分配、盲法设置、统计分析方法等进行质量评价，计分1分-5分，1分-2分为低质量，3分-5分为高质量。

### 1.5 统计学处理

采用Cochrane协作网提供的RevMan 5.0统计软件进行*meta*分析。计数资料采用优势比（odds ratio, OR）为疗效分析统计量，计量资料采用均数差（mean difference, MD）。各效应量均以95%可信区间（confidence interval, CI）表示。各纳入研究结果间的异质性采用*Chi^2^*检验。当各研究间无统计学异质性（*P* > 0.1, *I^2^* < 50%），采用固定效应模型对各研究进行*meta*分析^[18]^；如各研究间存在统计学异质性（*P* < 0.1, *I^2^* > 50%），分析其异质性来源，对可能导致异质性的因素进行亚组分析，若两项研究组之间存在统计学异质性而无临床异质性或差异无统计学意义时，采用随机效应模型进行分析。

## 2 结果

### 2.1 纳入研究的基本特征

按照检索策略初步检索文献后，按纳入标准经过阅读全文提取出5项合格的随机对照试验（表 1），其中2项研究^[3, 16]^为凡德他尼+多西他赛*vs*多西他赛，1项研究^[19]^为凡德他尼*vs*吉非替尼，1项研究^[9]^为凡德他尼*vs*厄洛替尼，1项研究^[20]^为凡德他尼+培美曲塞*vs*培美曲塞，总共包括3, 416例病例。4项研究^[3, 9, 19, 20]^报告了双盲，5项研究^[3, 9, 16, 19, 20]^均采用了随机方法。对5项研究进行评分，所有文献均在3分-5分，故最终纳入*meta*分析。

**1:** 纳入研究的基本特点 Basic characteristic of included studies

First author	Year	Stage of NSCLC	Vandetanib group	Placebo group	Jadad score
Heymach^[16]^	2007	Ⅲb or Ⅳ	vandetanib+docetaxel	docetaxel	3
Natale^[19]^	2009	Ⅲb or Ⅳ	vandetanib	gefitinib	4
Herbst^[3]^	2010	Ⅲb or Ⅳ	vandetanib+docetaxel	docetaxel	4
Natale^[9]^	2011	Ⅲb or Ⅳ	vandetanib	erlotinib	3
de Boer RH^[20]^	2011	Ⅲb or Ⅳ	vandetanib+pemetrexed	pemetrexed	4
NSCLC: non-small cell lung cancer.					

### 2.2 *meta*分析

#### 2.2.1 有效性（图 1）

**1:**
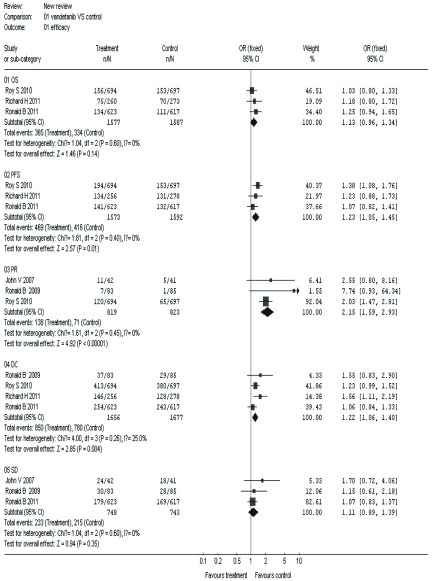
凡德他尼组与对照组治疗晚期NSCLC总生存期、疾病无进展生存期、部分缓解、疾病控制、疾病稳定比较 The outcome of OS, PFS, PR, DC, SD in the vandetanib versus control for advanced non-small cell lung cancer (NSCLC). OS: overall survival; PFS: progression free survival; PR: partial responses; DC: disease control; SD: stable disease.

##### 2.2.1.1 OS

3项研究^[3, 9, 20]^报道了OS，各研究间无统计学异质性（*P*=0.60, *I^2^*=0%），采用固定效应模型进行*meta*分析，结果显示两组在OS方面的差异无统计学意义（OR=1.13, 95%CI: 0.96-1.34）。

##### 2.2.1.2 PFS

3项研究^[3, 9, 20]^报道了PFS，各研究间无统计学异质性（*P*=0.40, *I^2^*=0%），采用固定效应模型进行*meta*分析，结果显示两组在PFS方面的差异有统计学意义（OR=1.23, 95%CI: 1.05-1.45）。

##### 2.2.1.3 PR

4项研究^[3, 9, 16, 20]^报道了PR，各研究间存在统计学异质性（*P*=0.02, *I^2^*=71.2%），经敏感性分析排除1篇文献^[9]^后各研究间无统计学异质性（*P*=0.45, *I^2^*=0%），采用固定效应模型进行*meta*分析，结果显示两组在PR方面的差异有统计学意义（OR=2.15, 95%CI: 1.59-2.93）。

##### 2.2.1.4 DC

4项研究^[3, 9, 19, 20]^报道了DC，各研究间无统计学异质性（*P*=0.26, *I^2^*=25.0%），采用固定效应模型进行*meta*分析，结果显示两组在DC方面的差异有统计学意义（OR=1.22, 95%CI: 1.06-1.40）。

##### 2.2.1.5 SD

3项研究^[16, 19, 20]^报道了SD，各研究间无统计学异质性（*P*=0.60, *I^2^*=0%），采用固定效应模型进行*meta*分析，结果显示两组在SD方面的差异无统计学意义（OR=1.11, 95%CI: 0.89-1.39）。

#### 2.2.2 安全性（图 2）

**2:**
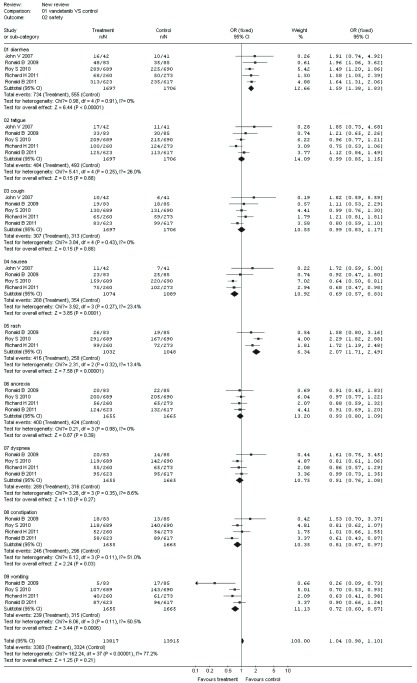
凡德他尼组与对照组治疗晚期NSCLC腹泻、疲乏、咳嗽、恶心、皮疹、食欲减退、呼吸困难、便秘、呕吐发生率的比较 The outcome of diarrhea, fatigue, cough, nausea, rash, anorexia, dyspnea, constipation, vomiting in the vandetanib versus control for advanced NSCLC

##### 2.2.2.1 腹泻

5项研究^[3, 9, 16, 19, 20]^报道了腹泻，各研究间无统计学异质性（*P*=0.91, *I^2^*=0%），采用固定效应模型进行*meta*分析，结果显示两组在腹泻方面的差异有统计学意义（OR=1.59, 95%CI: 1.38-1.83）。

##### 2.2.2.2 疲乏

5项研究^[3, 9, 16, 19, 20]^报道了疲乏，各研究间无统计学异质性（*P*=0.25, *I^2^*=26%），采用固定效应模型进行*meta*分析，结果显示两组在疲乏方面的差异无统计学意义（OR=0.99, 95%CI: 0.85-1.15）。

##### 2.2.2.3 咳嗽

5项研究^[3, 9, 16, 19, 20]^报道了咳嗽，各研究间无统计学异质性（*P*=0.43, *I^2^*=0%），采用固定效应模型进行*meta*分析，结果显示两组在咳嗽方面的差异无统计学意义（OR=0.99, 95%CI: 0.83-1.17）。

##### 2.2.2.4 恶心

5项研究^[3, 9, 16, 19, 20]^报道了恶心，各研究间存在统计学异质性（*P*=0.01, *I^2^*=67.8%），经敏感性分析排除1篇文献^[9]^后，各研究间无统计学异质性（*P*=0.27, *I^2^*=23.4%），采用固定效应模型进行*meta*分析，结果显示两组在恶心方面的差异有统计学意义（OR=0.69, 95%CI: 0.57-0.83）。

##### 2.2.2.5 皮疹

4项研究^[3, 9, 19, 20]^报道了皮疹，各研究间存在统计学异质性（*P* < 0.000, 01, *I^2^*=95.0%），经敏感性分析排除1篇文献^[9]^后，各研究间无统计学异质性（*P*=0.32, *I^2^*=13.4%），采用固定效应模型进行*meta*分析，结果显示两组在恶心方面的差异有统计学意义（OR=2.07, 95%CI: 1.71-2.49）。

##### 2.2.2.6 食欲减退

4项研究^[3, 9, 19, 20]^报道了食欲减退，各研究间无统计学异质性（*P*=0.98, *I^2^*=0%），采用固定效应模型进行*meta*分析，结果显示两组在食欲减退方面的差异无统计学意义（OR= 0.93, 95%CI: 0.80-1.09）。

##### 2.2.2.7 呼吸困难

4项研究^[3, 9, 19, 20]^报道了呼吸困难，各研究间无统计学异质性（*P*=0.35, *I^2^*=8.6%），采用固定效应模型进行*meta*分析，结果显示两组在呼吸困难方面的差异无统计学意义（OR=0.91, 95%CI: 0.76-1.08）。

##### 2.2.2.8 便秘

4项研究^[3, 9, 19, 20]^报道了便秘，各研究间无统计学异质性（*P*=0.11, *I^2^*=51%），采用固定效应模型进行*meta*分析，结果显示两组在便秘方面的差异有统计学意义（OR=0.81, 95%CI: 0.67-0.97）。

##### 2.2.2.9 呕吐

4项研究^[3, 9, 19, 20]^报道了呕吐，各研究间无统计学异质性（*P*=0.11, *I^2^*=50.5%），采用固定效应模型进行*meta*分析，结果显示两组在呕吐方面的差异有统计学意义（OR=0.72, 95%CI: 0.60-0.87）。

#### 2.2.3 发表偏倚

研究中采取了以下策略尽量减少发表偏倚：①通过严谨的检索策略搜索符合标准的文献；②制定排除标准对纳入文献进行筛选；③采用漏斗图对文献潜在的发表偏倚进行检验，漏斗图（图 3）显示基本对称，提示发表偏倚的可能性较小。

**3:**
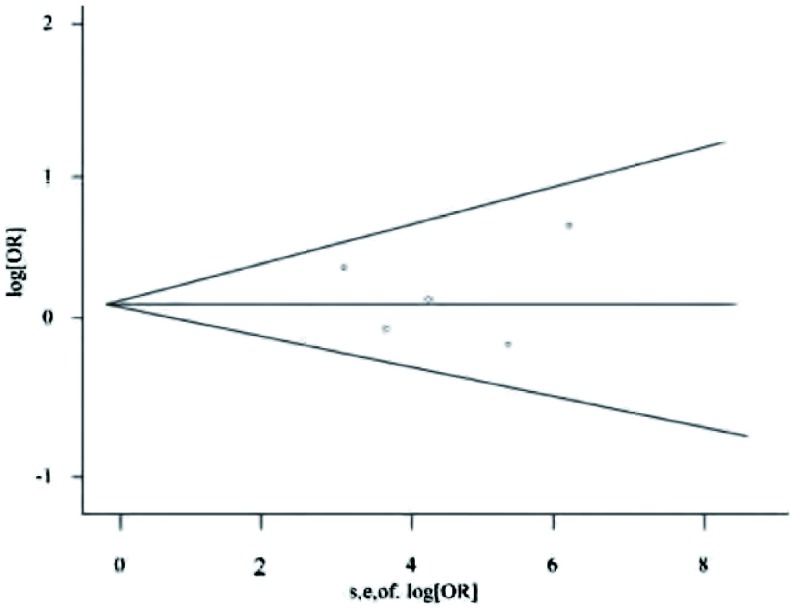
检验发表偏倚的漏斗图 Funnel plot of publication bias

## 3 讨论

本研究通过对国外凡德他尼作为二线方案治疗NSCLC的随机对照试验的*meta*分析，系统地评价了凡德他尼的有效性和安全性。结果显示，相对于单用一种其它靶向药物或化疗药物而言，凡德他尼可相对延长疾病PFS和提高PR和DC，而OS、SD率等方面无优势；凡德他尼的不良反应未见明显优势，主要表现为腹泻、皮疹相对较高，而恶心、便秘、呕吐相对较低，其它如疲乏、咳嗽、食欲减退、呼吸困难等无明显差异。

凡德他尼是一类口服EGFR、VEGFR及RET信号通路多靶点抑制剂。一项关于凡德他尼联合多西他赛的随机Ⅲ期临床研究^[3]^表明，凡德他尼联合多西他赛可延长PFS（4.0个月*vs* 3.2个月，*P* < 0.001）与客观缓解率（17% *vs* 10%, *P* < 0.001）。根据FACT-L肺癌量尺，症状恶化时间在凡德替尼组中也有明显改善（*P* < 0.001）。凡德他尼是第一个在Ⅲ期临床试验^[3]^中被证实与标准化疗方案联合能达到明显临床受益的口服靶向药物。本研究纳入的5项研究^[3, 9, 16, 19, 20]^中有2项研究^[9, 19]^是单用凡德他尼治疗晚期NSCLC，各研究间尚存在一定的异质性，容易产生文献选择偏倚。因本研究纳入的文献数量少且都为国外文献，可能会产生发表偏倚。发表偏倚最常见的原因是大样本阳性结论文章发表快，小样本阴性研究发表慢，而阴性结论的研究常常被拒绝发表，发表偏倚有时是不可避免的。只有通过严格试验设计、多国多中心的大规模临床试验才能尽量减少发表偏倚。分析中部分缓解、恶心、皮疹等结果异质性较大，经敏感性分析剔除了引起异质性产生的文献后同质性较好，使结果具有一定的稳定性。但样本量的减少对其结论的可靠性有一定程度的影响，故在以后的研究中需要更多大样本、同质性较好的随机对照试验加以论证。

纳入的5项研究^[3, 9, 16, 19, 20]^均是随机对照试验。纳入研究均提及采用了随机分配，1项研究^[3]^采用了分配隐藏方法，其余4项^[9, 16, 19, 20]^均未提及分配隐藏。4项研究^[3, 9, 19, 20]^采用了双盲法，1项研究^[16]^未提及盲法，有研究显示即使采用了正确的随机方法，但如果未对随机序列进行有效隐藏，依然会在纳入患者时产生选择偏倚，影响研究结果的真实性。因此，希望今后的研究在随机双盲试验的基础上，实施分配隐藏，并报道失访情况，以期进一步提高研究质量。

综上所述，凡德他尼作为二线方案治疗NSCLC在一定程度上可以延长疾病PFS并提高PR和DC，达到短期效应。但其对应的不良反应，仍需临床予以有效的对症处理。由于纳入研究的局限性，凡德他尼的远期结果还不明确，希望在以后的临床随机对照试验中详细报道整体生存期和生活质量，进行更长时间的随访观察并报道详细的终点指标，以进一步验证其长期的有效性和安全性。
